# Comparison of LRINEC Scoring System with Finger Test and Histopathological Examination for Necrotizing Fasciitis

**DOI:** 10.1055/s-0041-1740629

**Published:** 2022-01-17

**Authors:** Farah Naaz Kazi, J.V. Sharma, Shaurav Ghosh, D. Prashanth, V. Om Pramod Kumar Raja

**Affiliations:** 1 Department of General Surgery, Vydehi Institute of Medical Sciences and Research Centre, Bangalore, Karnataka, India

**Keywords:** necrotizing fasciitis, LRINEC, Finger test, histopathology

## Abstract

**Background** Necrotizing fasciitis (NF) is a life-threatening condition requiring urgent attention. It is clinically difficult to diagnose, linked to severe systemic toxicity, and has poor prognosis. In 2001, Andreasen and coworkers described the “Finger test” for the diagnosis of NF. Subsequent studies have suggested early recognition and management of NF. In this study, we compare the LRINEC—Laboratory Risk Indicator for Necrotizing Fasciitis—scoring system with the “Finger test” and histopathological examination for diagnosis of NF.

**Results** In our study, LRINEC scoring system and Finger test are statistically significant in the diagnosis of NF. Males are more frequently affected, and the most common organism causing NF is *Staphylococcus* . Histopathology remained the gold standard for diagnosis of NF, while LRINEC score and Finger test were good diagnostic tools for early diagnosis, with sensitivities of 83.33 and 86.11%, respectively.

**Conclusion** LRINEC laboratory-based scoring system is easy and reliable diagnostic tool though histopathology remains the gold standard. There is statistically significant correlation between histopathology and laboratory criteria. LRINEC test is independently better than bedside Finger test alone or combined LRINEC and bedside Finger test.

Necrotizing fasciitis (NF) is a rapidly progressing fascial tissue inflammation and necrosis, with relative skin and underlying muscle sparing. A surgical emergency managed by patient optimization is the key to achieve high quality post operative results.

There is a stark difference between inflamed tissue and necrotized tissue. Inflamed tissue is the damaged tissue in response to the microorganism or injury, which leads to an increased blood supply and permeability of blood vessels, while necrotized tissue consists of dead and decaying group of cells caused by infection, trauma, or toxins, which in turn delays the healing of the tissues.

The average annual incidence of NF for every 100,000 inhabitants was 0.86. 1 With respect to all age groups, the incidence of NF spiked to 2.5 times more for men across all age groups. 1 Among the comorbid patients, diabetic individuals were found to be more affected. *Clostridium* infections also are major causative factor. Delay in surgical intervention can be fatal. Another pitfall in diagnosis of NF is that even patients with mild pain, absence of fever, and crepitus can have soft tissue infection. Diagnostic tools used in determining NF are Laboratory Risk Indicator for Necrotizing Fasciitis (LRINEC) scoring system, Finger test, histopathology tissue culture, and radiological investigations. LRINEC scoring system is a sum total of C-reactive protein (CRP) test, total count, serum sodium, serum creatinine, and blood glucose levels. 2 Positive and negative predictive values of 92% and 96%, respectively, are found when the LRINEC score is 6 or more. A value of <5 is considered low risk, 6 to 7 is considered of intermediate risk, while > 8 is considered high risk. In case of ambiguity regarding diagnosis, Finger test was done, which was proposed by Andreasen et al. 3 Pointers of NF are lack of bleeding, appearance of malodorous dish water pus, and lack of finger dissection resistance. 2

Stamenkovic and Lew 4 showed that early frozen tissue biopsy can provide a conclusive and life-saving diagnosis in a case of suspected NF. McHenry et al 5 showed that early necrotizing soft tissue injury (NSTI) debridement was linked to a large mortality decline. Bilton et al 6 showed that vigorous and immediate operative intervention of NF decreased the number of deaths.

*Aim:* Comparative study between LRINEC scoring system, Finger test, and histopathology for discerning NF from other soft tissue infections.

## Materials and Methodology

### Source of Data

All patients admitted to the General Surgery Outpatient Department of a tertiary care hospital in Bangalore were considered. Sample sizes of 40 patients were taken. Study duration of 18 months, with a follow-up period of 6 months, was considered.

### Inclusion Criteria

Patients clinically diagnosed with NF, belonging to the age group of 18 to 90 years of either gender were included.

### Exclusion Criteria

Patients previously diagnosed with NF were not considered. Patients on steroid treatment or with deep vein thrombosis, abscess, erysipelas, lymphedema, peripheral vascular disease, ischemic heart disease, and cerebrovascular accidents were excluded. Only patients willing to consent to the study were included. Patients not having enough background investigations, hence LRINEC score cannot be calculated, and patients who have already participated in the study and have come for a follow-up were also excluded.

### Study Design

An observational study.

### Methods and Data Collection

Clinical evaluations and investigations involving 40 patients diagnosed with soft tissue infections. LRINEC tests, Finger test, and histopathology examinations were done. Data were collected and statistical analysis done. With regard to statistical methods, descriptive and inferential statistical analysis was executed in the current research. Mean ± standard deviation (SD; Min-Max) represented the continuous measurements while the number (%) represented the categorical measurements. A 5% level of significance was used to evaluate the significance. Fisher's exact test/chi-squared test was used to discover the relevance of research parameters on categorical scale between two or more groups. Qualitative data analysis used the nonparametric setting. For the small cell samples, the Fisher's exact test was worked with. Suggestive significance had a *p* -value between 0.05 and 0.10, moderately significant had a *p* -value between 0.01 and 0.05, while for strongly significant, the *p* -value was ≤0.01.

To calculate the specificity and sensitivity of the criteria, data were distributed according to the outcome of the diagnosis. Sensitivity measures the proportion of actual positives that are correctly identified as such, that is, the percentage of study group that was correctly identified to have NF. Specificity measures the proportion of actual negatives that are correctly identified as such, that is, the percentage of study group that was correctly identified to not have NF.

The ethics approval was obtained from Sri Manakula Vinayagar Medical College and Hospital, Pondicherry, while the data compilation was done in Vydehi Institute of Medical Sciences and Research Centre, Bangalore.

## Results

Comparison of various parameters of LRINEC score in Table 1 suggests that patients were in age 57.05 ± 13.64 (SD) with *p* = 0.435, insignificant. Hemoglobin (g/dL) was 10.52 ± 2.19 (SD) with *p* = 0.33, insignificant. Total leukocyte count (TLC) was around 19,300 ± 7,065.19 (SD) with *p* = 0.032. Serum creatinine was 180.25 ± 138.85 (SD) with *p* = 0.020, significant. Serum sodium was 135 ± 5.57 (SD) with *p* = 0.056, significant. Serum glucose was 13.54 ± 8.98 (SD) with *p* = 0.111, insignificant. CRP was 162.45 ± 46.10 (SD) with *p* < 0.001, very highly significant.

**Table 1:** Comparison of clinical variables according to LRINEC results

Variables	LRINEC result		Total	*p* -Value
	Negative	Positive		
Age	60.22 ± 13.89	56.12 ± 13.66	57.05 ± 13.64	0.435
HB (g/dL)	11.15 ± 2.61	10.33 ± 2.07	10.52 ± 2.19	0.333
TLC (/cu.mm)	14,900.00 ± 6,523.80	20,577.41 ± 67,985.90	19,300.00 ± 7,065.19	0.032*
Serum creatinine (micmol/L)	86.66 ± 20.00	207.41 ± 146.85	180.25 ± 138.85	0.020*
Serum sodium (mmol/L)	138.11 ± 4.85	134.09 ± 5.50	135.00 ± 5.57	0.056+
Serum glucose (mmol/L)	9.33 ± 3.66	14.76 ± 9.72	13.54 ± 8.98	0.111
C-reactive protein (mg/L)	97.55 ± 20.70	181.29 ± 31.91	162.45 ± 46.10	<0.001**

Abbreviations: HB, hemoglobin; LRINEC, laboratory risk indicator for necrotizing fasciitis; TLC, total leukocyte count.

* Significant values.

In Table 2 , total 40 patients (100%) were considered. Finger test and LRINEC results were both positive in 28 patients (90.3%). Positive predictive value (PPV) = 90.32 with sensitivity = 82.35, specificity = 50.00, *p* -value = 0.080 + , and accuracy = 77.50. Also, considering histopathology tissue culture and LRINEC results, 30 patients (96.8%) were positive. Total 36 (90%) patients confirmed necrotized tissue, remaining 4 (10%) confirmed inflamed tissue. PPV = 96.77, sensitivity = 83.33, specificity = 75.00, *p* -value = 0.008**, and accuracy = 82.58.

**Table 2:** Finger test and histopathology distribution in relation to LRINEC results in patients studied

Bedside Finger test	LRINEC result		Total
	Negative	Positive	
Negative	3 (33.3%)	3 (9.7%)	6 (15%)
Positive	6 (66.7%)	28 (90.3%)	34 (85%)
Total	9 (100%)	31 (100%)	40 (100%)
**Histopathology**	LRINEC result		Total
	Negative	Positive	
Necrotized tissues	6 (66.7%)	30 (96.8%)	36 (90%)
Inflamed tissue	3 (33.3%)	1 (3.2%)	4 (10%)
Total	9 (100%)	31 (100%)	40 (100%)

Abbreviation: LRINEC, laboratory risk indicator for necrotizing fasciitis.

### Chi-Squared Test/Fisher's Exact Test

In Table 3 , the most common microorganism isolated comparing pus culture and sensitivity and LRINEC results is *Staphylococcus* . Total 15 (37.55%) out of 40 patients were diagnosed positive for the same. Of these, 1 (11.1%) was LRINEC negative and 14 (45.2%) were LRINEC positive; *p* = 0.117, insignificant. *Streptococcus* was detected in total of nine (22.5%) patients. Of these, five (55.6%) were LRINEC negative and four (12.9%) were positive; *p* = 0.016, significant.

**Table 3:** Pus C/S distribution in relation to LRINEC and histopathology results in patients studied

Pus C/S	LRINEC result		Total ( *n* = 40)	*p* -Value	Histopathology		Total ( *n* = 40)	*p* -Value
	Negative ( *n* = 9)	Positive ( *n* = 31)			Inflamed tissue ( *n* = 4)	Necrotized tissues ( *n* = 36)		
*Acinetobacter*	0 (0%)	2 (6.5%)	2 (5%)	1.000	0 (0%)	2 (5.6%)	2 (5%)	1.000
*Diphtheroid commensals*	0 (0%)	1 (3.2%)	1 (2.5%)	1.000	0 (0%)	1 (2.8%)	1 (2.5%)	1.000
*Escherichia coli*	1 (11.1%)	5 (16.1%)	6 (15%)	1.000	0 (0%)	6 (16.7%)	6 (15%)	1.000
*Methicillin-resistant Staphylococcus aureus*	1 (11.1%)	0 (0%)	1 (2.5%)	0.225	1 (25%)	0 (0%)	1 (2.5%)	0.100
*Proteus mirabilis*	1 (11.1%)	2 (6.5%)	3 (7.5%)	0.545	0 (0%)	3 (8.3%)	3 (7.5%)	1.000
*Pseudomonas aeruginosa*	0 (0%)	3 (9.7%)	3 (7.5%)	1.000	1 (25%)	2 (5.6%)	3 (7.5%)	0.277
*Staphylococcus*	1 (11.1%)	14 (45.2%)	15 (37.5%)	0.117	0 (0%)	15 (41.7%)	15 (37.5%)	0.278
*Streptococcus*	5 (55.6%)	4 (12.9%)	9 (22.5%)	0.016*	2 (50%)	7 (19.4%)	9 (22.5%)	0.213

Abbreviations: C/S, culture and sensitivity; LRINEC, laboratory risk indicator for necrotizing fasciitis.

Also, comparing pus culture and sensitivity with histopathology tissue culture results, it has showed *Staphylococcus* in 15 (37.5%) out of 40 patients; *p* = 0.278, insignificant. *Streptococcus* was detected in 9 (22.5%) of 40 patients.

### Chi-Squared Test/Fisher's Exact Test

In Table 4 , age and gender distribution in relation to LRINEC results showed the following. Of 15 patients (37.5%) considered, the positive 13 patients (41.9%) belonged to 61 to 70 years age group. Majority of the patients positive for LRINEC were male (80.6%). Also majority of patients positive for histopathology were males (77.8%).

**Table 4:** Frequency distribution of age and gender distribution in relation to LRINEC and histopathology results in patients studied

Variables	LRINEC result		Total	*p* -Value	Histopathology		Total	*p* -Value
	Negative	Positive			Inflamed tissue	Necrotized tissues		
Age								
≤40	0 (0%)	4 (12.9%)	4 (10%)	0.247	0 (0%)	4 (11.1%)	4 (10%)	0.658
41–50	3 (33.3%)	6 (19.4%)	9 (22.5%)		2 (50%)	7 (19.4%)	9 (22.5%)	
51–60	2 (22.2%)	7 (22.6%)	9 (22.5%)		0 (0%)	9 (25%)	9 (22.5%)	
61–70	2 (22.2%)	13 (41.9%)	15 (37.5%)		2 (50%)	13 (36.1%)	15 (37.5%)	
> 70	2 (22.2%)	1 (3.2%)	3 (7.5%)		0 (0%)	3 (8.3%)	3 (7.5%)	
Gender								
Female	2 (22.2%)	6 (19.4%)	8 (20%)	1.000	0 (0%)	8 (22.2%)	8 (20%)	0.566
Male	7 (77.8%)	25 (80.6%)	32 (80%)		4 (100%)	28 (77.8%)	32 (80%)	
Total	9 (100%)	31 (100%)	40 (100%)		4 (100%)	36 (100%)	40 (100%)	

Abbreviation: LRINEC, laboratory risk indicator for necrotizing fasciitis.

In Table 5 , comparing bedside Finger test and histopathology, out of total 34 patients (85%, *n* = 100), 31 patients (86.1%) were positive for both bedside Finger test and histopathology findings: *p* = 0.554, insignificant; PPV = 91.18; sensitivity = 86.11, specificity = 25.00; and accuracy = 80.00. Also, comparing LRINEC results with histopathology, out of total 31 patients (77.5%, *n* = 100), 30 patients (83.3%) were positive for both LRINEC results and histopathology findings: *p* = 0.008**, significant; PPV = 96.77; sensitivity = 83.33; specificity = 75.00; and accuracy = 82.58.

**Table 5:** Association of Finger test and LRINEC results in association with histopathology findings

Bedside Finger test	Histopathology		Total
	Inflamed tissue	Necrotized tissues	
Negative	1 (25%)	5 (13.9%)	6 (15%)
Positive	3 (75%)	31 (86.1%)	34 (85%)
Total	4 (100%)	36 (100%)	40 (100%)
**LRINEC result**	Histopathology		Total
	**Inflamed tissue**	**Necrotized tissues**	
Negative	3 (75%)	6 (16.7%)	9 (22.5%)
Positive	1 (25%)	30 (83.3%)	31 (77.5%)
Total	4 (100%)	36 (100%)	40 (100%)

Abbreviation: LRINEC, laboratory risk indicator for necrotizing fasciitis.

### Chi-Squared Test/Fisher's Exact Test

Table 6 presents association of LRINEC results with histopathology findings and LRINEC results. Of the 31patients (100%), 28 (90.3%) were positive for both: *p* = 0.024. Studies of LRINEC results showed that out of 34 patients (100%), 28 (82.4%) were positive for LRINEC results with histopathology findings and LRINEC results: *p* = 0.115. Hence association between the two.

**Table 6:** Association of LRINEC results with the histopathology findings and LRINEC results

Histopathology	Finger test		Total	*p* -Value
	Negative	Positive		
**Inflamed tissue**				
LRINEC result				
➢ Negative	0 (0%)	3 (100%)	3 (75%)	0.250
➢ Positive	1 (100%)	0 (0%)	1 (25%)	
➢ Total	1 (100%)	3 (100%)	4 (100%)	
**Necrotized tissues**				
LRINEC result				
➢ Negative	3 (60%)	3 (9.7%)	6 (16.7%)	0.024*
➢ Positive	2 (40%)	28 (90.3%)	30 (83.3%)	
➢ Total	5 (100%)	31 (100%)	36 (100%)	
**Total**				
LRINEC result				
➢ Negative	3 (50%)	6 (17.6%)	9 (22.5%)	0.115
➢ Positive	3 (50%)	28 (82.4%)	31 (77.5%)	
➢ Total	6 (100%)	34 (100%)	40 (100%)	

Abbreviation: LRINEC, laboratory risk indicator for necrotizing fasciitis.

In Table 7 , association of LRINEC + Finger test results with histopathology findings was studied. Of the 36 patients (100%), 33 (91.7%) were positive for histopathology findings and LRINEC + Finger test: *p* = 1.000.

**Table 7:** Association of LRINEC + bedside Finger test results with histopathology findings

LRINEC result + Finger test	Histopathology		Total
	Inflamed tissue	Necrotized tissues	
Negative	0	3 (8.3%)	3 (7.5%)
Positive	4 (100.0%)	33 (91.7%)	37 (92.5%)

Abbreviation: LRINEC, laboratory risk indicator for necrotizing fasciitis.

*p* = 1.000; Significant, Fisher's Exact Test

## Discussion

McHenry et al 5 reported that prompt and quick debridement correlated with significant decreased mortality. *Streptococcus pyogenes* infection was the most common cause of mono-microbial NSTI, but was not linked to increased deaths. In our study, the commonest microorganism isolated was *Staphylococcus* . Wong et al 7 showed LRINEC score detected NF in its early clinical stages. The presence of NF in patients with LRINEC score of ≥ 6 should be thoroughly assessed for. In our study, hematological changes ( Table 3 ) in NF are consistent with any septic process. Severe anemia and systemic sepsis are not contraindications for surgery. Patient optimization decreases presurgical risk factors. Serum sodium ( Table 5 ) was significantly lower in patients diagnosed with NF. These results may characterize kidney dysfunction due to multiorgan infection damage and also hyponatremia due to fluid sequestration in serious soft tissue infections. An elevated glycemic gap was significantly independently associated with outcomes in diabetic NF patients ( Table 6 ). We also concluded ( Table 7 ) that CRP and creatinine were higher in patients with NF suggesting ( Table 4 ) increased risk of morbidity in patients diagnosed with NF.

Taviloglu et al 8 showed that patients of age more than 55 years with perineal localization were individual risk factors for idiopathic NF. Polymicrobial infections seem the most predominant (82%) while the death rate was found to be 35% with predominance among women, and patients with malignant diseases and diabetes mellitus. Stevens and Bryant 9 showed that early diagnosis, surgical intervention, and appropriate antibiotic treatment reduce mortality and improve outcomes. In our study, elderly patients constitute a risk factor for higher incidence and morbidity ( Table 1 ). This increases with risk factors like renal failure or delayed surgical debridement. It is also related to progression of the disease and a more severe infection. Majority of NF patients in our research were males ( Table 2 ). Length of hospital stay and the number of deaths of NF are similar in both the genders.

Stevens et al 10 recommended guidelines emphasizing clinical skills to diagnose skin and soft tissue infections promptly, detect pathogens, and deliver effective treatments at the earliest. Group A *streptococci (S. pyogenes)* cause severe invasive infections. Anaya and Dellinger 11 showed that early and full surgical debridement along with antimicrobial treatment and close monitoring shall be the heart of the treatment. The 2004 LRINEC score was initially released based on routine parameters and provides a method to detect cases at an early stage. Golger et al 12 showed that age, streptococcal toxic shock syndrome, and immune status are important mortality determinants and may, shortly after admission, predict death from NF. In our study also, early diagnosis and aggressive treatment remain key to management and we concur to this.

Lee et al 13 showed that glycopeptides can be used as an empirical therapy in patients with severe invasive NF caused by community-associated methicillin-resistant *Staphylococcus aureus* until the susceptibility results are available. Its prognosis along with surgical intervention was found to be excellent. Su et al 14 concluded that the LRINEC score is linked with the outcomes of patients with NSTI. The death rates and amputation are higher in patients with ≥6 LRINEC score. Frazee et al 15 concluded that pathologically defined NSTIs are present with a vast range of symptoms and early diagnosis is challenging. Hsiao et al 16 showed that in patients with NF, the independent positive predictors of death rate are *Vibrio* and *Aeromonas* infection, cancer, hypotension, and band form white blood cell count greater than 10%. On the other hand, *Streptococcal* and *Staphylococcal* infections are not predictors of death rate. Hemorrhagic bulla is an autonomous negative predictor of mortality. Cheung et al 17 concluded that clinicians should have high index of suspicion for NF and should start empirical therapy with repeated clinical tests. High survival rates are observed when surgical intervention of first fasciotomy and radical debridement are done within 24 hours of appearance of the symptoms.

Puvanendran et al 2 emphasized that safeguarding must be done while treating patients with erythema, pain, and fever to make sure that this life-threatening condition is not missed. Hsu et al 18 showed that clinicians should be warned to differentiate between NF infections with *Vibrio vulnificus* (contact with seawater or raw seafood) and *Klebsiella pneumoniae* (abrasions or chronic ulcers) in diabetic patients based on exposure history and hospital presentation. *V. vulnificus* infections are more infectious than *K. pneumoniae* infections during the early stage.

Misiakos et al 19 showed that early and aggressive drainage and debridement remains mainstay of treatment. Bryant and Stevens (2015) showed the need for early aggressive management of *S. pyogenes* infection. 20 Pasternack and Swartz 21 showed that lack of early diagnosis led to increased morbidity and mortality apart from escalated cost of treatment.

Khamnuan et al 22 showed that risk factors of mortality in patients with NF included being female, age >60 years, skin necrosis, pulse rate >130/min, systolic blood pressure <90 mm Hg, having chronic heart disease, liver cirrhosis, and serum creatinine level ≥1.6 mg/dL. Therefore, patients with the above must undergo a serious surveillance to prevent further complications. Shaikh et al 23 concluded that although males have a tendency to develop NF, females may also be at a high risk as they can develop NF of the groin and abdominal wall.

Organ failure is one of the most common complications seen in males. NF continues to be challenging as the mortality rate fluctuates between 25 and 27% among both male and female.

Bechar et al 24 concluded that LRINEC score is a useful tool for patients with NF. El-Menyar et al 25 stated that the scoring system would identify potential patients with NF along with pointing out hospital outcomes. Misiakos et al 26 showed that clinicians with a close watch for NF showed better survival rates. Latifi et al 27 concluded that time to surgery is a significant independent predictor of length of hospital stay.

In our research, we discovered that the majority of patients with histologically proven NF had an LRINEC score of 6 or higher. Our findings also proved that a combination of LRINEC scoring, Finger test, and histopathology tissue culture seemed to be very effective in diagnosing NF. *S. aureus* is the most common organism cultured from pus cultures. *Streptococcus* was the second most commonly cultured organism. Among the various parameters in LRINEC, TLC, serum creatinine, and CRP proved to have significant *p* -values in our study.

### Limitations

Considering the fact that NF is a rare disease, the study was limited only to a small sample of patients. Another major limitation was the inability to calculate all the parameters of LRINEC for all patients. Hence, to overcome this, patients were added from low-risk NF as well and extensive search was done on our patient database to collect all the values.

## Conclusion

LRINEC laboratory-based scoring system is an easy and reliable diagnostic tool for diagnosis of NF, though histopathology remains the gold standard for the diagnosis. In our study, we also found a statistically significant correlation between histopathology and laboratory criteria, and that LRINEC test is independently better than Finger test alone or combined LRINEC and Finger test. Also, patients with concomitant diseases showed poor prognosis.
